# Resilience in patients with complex regional pain syndrome 1—a cross-sectional analysis of patients participating in a cross-sectional cohort study

**DOI:** 10.1093/pm/pnad055

**Published:** 2023-05-08

**Authors:** Maria Monika Wertli, Barbara Aegler, Candida S McCabe, Sharon Grieve, Alison Llewellyn, Stephanie Schneider, Lucas M Bachmann, Florian Brunner

**Affiliations:** Department of General Internal Medicine, Cantonal Hospital Baden, Baden 5404, Switzerland; Department of Internal Medicine, University Hospital Bern, Bern University, Bern 3010, Switzerland; Praxis für Handrehabilitation und Ergotherapie GmbH, Zurich 8008, Switzerland; Dorothy House Hospice, Winsley and University of the West of England, Bristol BS16 1QY, UK; Royal United Hospitals Bath NHS Foundation Trust, Bath BA1 3NG, UK; Faculty of Health and Applied Sciences, University of the West of England, Bristol BS16 1QY, UK; Faculty of Health and Applied Sciences, University of the West of England, Bristol BS16 1QY, UK; Department of Physical Medicine and Rheumatology, Balgrist University Hospital, Zurich 8004, Switzerland; Medignition Inc, Zurich 8008, Switzerland; Department of Physical Medicine and Rheumatology, Balgrist University Hospital, Zurich 8004, Switzerland

**Keywords:** complex regional pain syndrome, resilience, anxiety, fatigue, depression, fear avoidance, chronic pain, pain catastrophizing

## Abstract

**Objective:**

To assess the degree of resilience in patients with complex regional pain syndrome (CRPS) 1, to explore the relationship between resilience and patient-related outcome measurements and to describe a pattern of clinical manifestations associated with low resilience.

**Methods:**

This study presents a cross-sectional analysis of baseline information collected from patients enrolled in a single center study between February 2019 and June 2021. Participants were recruited from the outpatient clinic of the Department of Physical Medicine & Rheumatology of the Balgrist University Hospital, Zurich, Switzerland. We used linear regression analysis to explore association of resilience with patient reported outcomes at baseline. Furthermore, we explored the impact of significant variables on the low degree resilience using logistic regression analysis.

**Results:**

Seventy-one patients (females 90.1%, mean age 51.2 ± 12.9 years) were enrolled. There was no association between CRPS severity and the level of resilience. Quality of Life was positively correlated with resilience, as was pain self-efficacy. Pain catastrophizing was inversely correlated with the level of resilience. We observed a significant inverse association between anxiety, depression and fatigue and the level of resilience. The proportion of patients with a low resilience increased with higher level of anxiety, depression and fatigue on the PROMIS-29, without reaching statistical significance.

**Conclusion:**

Resilience seems to be an independent factor in CRPS 1 and is associated with relevant parameters of the condition. Therefore, caretakers may screen the current resilience status of CRPS 1 patients to offer a supplementary treatment approach. Whether specific resilience training modifies CRPS 1 course, requires further investigations.

## Introduction

Complex regional pain syndrome (CRPS) describes a variety of painful conditions that usually appear on a distal extremity within six weeks after an initiating noxious event such as trauma or surgery.^1^ Typically, signs and symptoms exceed the expected clinical course of the inciting event in magnitude and duration, often resulting in disproportionate pain and significant impairment. CRPS is subdivided into two subgroups: Type 1 without distinct major nerve damage and Type 2 with evidence of a major nerve lesion.^1^ The clinical presentation includes a widespread spectrum of manifestations including sensory, vasomotor, sudomotor, motor, and trophic changes.^2^ As a result of this clinical heterogeneity, the diagnosis is often delayed and solely based on clinical signs and symptoms (revised Budapest criteria).^3^ Since there is no underlying or causal cure, the therapy remains symptom-based and includes a variety of pharmacological, interventional, and rehabilitative options (such as psychology, physical therapy, and occupational therapy). Although benign trajectories have been reported, several prospective studies indicate an unfavorable prognosis with persistent symptoms after one year.^4–6^

According to the definition introduced by the American Psychological Association, resilience described as the process of adapting well in the face of adversity, trauma, tragedy, threats or significant sources of stress.^7^ Resilience represents a dynamic construct which may change over time as a function of development and individual interaction with the environment.^8^ Determinants of resilience include a host of biological, psychological, social, and cultural factors that interact with one another to determine how one responds to stressful experiences.^9^ Recently, the concept of resilience has received growing interest regarding its potential influence on health, well-being, and quality of life.^10^ Although the efficacy of resilience interventions remain controversial,^11^ several studies have indicated that increased resilience can be positively associated with various aspects including physical function, satisfaction in social roles and quality of life.^12^^,^^13^ In patients with chronic pain, resilience predicted quality of life after a functional restoration program^14^ and improved outcome during return to work programs.^15^ Therefore, it may be hypothesized that building resilience may be helpful in the treatment and support of chronic conditions such as CRPS. An adequate degree of resilience potentially helps patients with CRPS to cope with the challenging circumstances. To date, however, CRPS guidelines do not recommend measuring resilience in the standard clinical practice. Hence, little is known about the typical resilience status of patients with CRPS and the association with other clinical parameters which are obtained in practice. Therefore, this cross-sectional study, enrolling patients referred to one specialized CRPS center,^1^ assessed the degree of patients’ resilience using standardized methods,^2^ explored the relationship between the degree of resilience and patient-related outcome measurements, and^3^ sought to identify any pattern of clinical manifestations associated with low resilience.

## Methods

### Setting and participants

This is a cross-sectional analysis of baseline information of patients participating in a single center study, was conducted between February 2019 and June 2021. The participants were consecutively recruited from the outpatient clinic of the Department of Physical Medicine & Rheumatology of the Balgrist University Hospital, Zurich, Switzerland. FB per-formed the screening visit with possible participants. In the context of the global COVID-19 pandemic, no regular consultations were held during the lockdown in Switzerland between March 16, 2020, and April 26, 2020. Accordingly, we were not able to recruit participants during this period. Written informed consent was obtained from all participants. The data were obtained within the feasibility study of collecting data for CRPS clinical studies (COMPACT-Q) using a core measurement set.^16^ The set of CRPS related parameters was selected based on the international recommendations of Core Outcome Measures for complex regional PAin syndrome Clinical Trials (COMPACT).^16^^,^^17^ We obtained permission from the distributors or license holders, where applicable, to use the standardized questionnaires in the relevant languages for the purposes of the COMPACT-Q feasibility study.

The study was approved by the Ethics Committee of Zurich (BASEC: 2019–00619).

All participants were adults (age ≥ 18) who met the diagnostic criteria for CRPS 1 of the hand or the foot according to the revised Budapest criteria.^18^ Excluded were patients not proficient in the German language and patients with any mental health condition which may detrimentally impede study participation.

### Assessment of resilience

#### Connor-Davidson resilience scale (CD-RISC)

The degree of resilience was assessed by the CD-RISC^19^ (permission of the authors was obtained). The scale consists of 25 items, which are evaluated on a 5-point Likert scale ranging from 0 to 4: not true at all (0), rarely true,^1^ sometimes true,^2^ often true,^3^ and true nearly all of the time.^4^ The sum score results in a number between 0–100, and higher scores indicate higher resilience. In the framework of the validation study, reference scores for the following groups were calculated: a community sample (n = 577), primary care outpatients (n = 139), general psychiatric outpatients (n = 43), a clinical trial of generalized anxiety disorders (n = 25), and two clinical trials of post-traumatic stress disorder (n = 22 in both trials).^19^ Further details of the reference groups were not described in the publication.

### Clinical parameters of CRPS

#### CRPS Severity Score (CSS)

Disease activity was recorded by using the CRPS Severity Score (CSS), which was directly derived from the Budapest CRPS diagnostic criteria.^20^ The CSS is completed by a clinician or an experienced healthcare professional by scoring the presence or absence (coded 1/0) of 8 signs and 8 symptoms. Higher scores indicate greater CRPS severity (range 0–16).

#### PROMIS-29

The Patient Reported Outcomes Measurement Information System 29-item Health Profile (PROMIS-29) is a generic patient reported outcome measure to be used with the general population and with individuals living with chronic conditions.^21^ Promis-29 provides measures of health status that assess physical, mental, and social well–being. The questionnaire includes 28 items from seven domains (depression, anxiety, physical function, pain interference, fatigue, sleep disturbance, and ability to participate in social roles and activities) and a single item on pain intensity. According to current recommendations,^17^ a suicidal ideation was assessed using a single PROMIS item^22^ in this study. Each item has five response options (values 1 to 5), except for the pain intensity item which has eleven response options (values 0 to 10). PROMIS measures generate T-scores with a mean of 50 and standard deviation of 10 in a reference population. Based on large scale calibration testing the T-scores are interpreted within normal limits, mild, moderate, severe.^23^

#### Short-form McGill Pain Questionnaire-2 (SF-MPQ-2)

According to the previously stated recommendations, neuropathic pain qualities were captured using the six neuropathic items from the SF-MPQ-2.^24^ Each item was rated based on a 0–10 scale with 0 equal to no pain and 10 equals to the worst pain ever during the past week. The total score is calculated by summing the individual scores. Higher scores indicate more neuropathic pain (range 0–60).

#### Pain Catastrophizing Scale (PCS)

The Pain Catastrophizing Scale (PCS) is a 13-item self-report measure designed to assess catastrophic thinking related to pain among adults.^25^ People are asked to indicate the degree to which they have the above thoughts and feelings when they are experiencing pain using the 0 (not at all) to 4 (all the time) scale. A total score is calculated (ranging from 0 to 52). Higher scores indicate more pain catastrophizing. Scores <30 indicate a not problematic thinking and ≥30 represent problematic levels of catastrophic thinking.^25^

#### EuroQoL 5-dimension 5-level instrument (EQ-5D-5L)

The EQ-5D-5L is a generic health-related quality of life measure.^26^ It consists of two pages: the descriptive system and the Visual Analogue Scale (EQ-VAS). The EQ-5D-5 L descriptive system consists of five dimensions as follows: mobility, self-care, usual activities, pain/discomfort, and anxiety/depression. Each dimension in the EQ-5D-5 L has five response levels: no problems (Level 1); slight; moderate; severe; and extreme problems (Level 5). A total of 3125 health states are defined for EQ-5D-5 L. Health states are from 1–1-1–1-1 (the best health state) to 5–5-5–5-5 (the worst health state). EQ-5D-5 L health states are converted into a single index “utility” score using a scoring algorithm. The instrument also includes a visual analogue scale (EQ-VAS) which provides a single global rating of self-perceived health and is scored on a 0 to 100 mm scale representing “the worst …” and “the best health you can imagine”, respectively.

#### Pain Self-Efficacy Questionnaire (PSEQ)

The PSEQ is a 10-item questionnaire developed to assess the confidence people with ongoing pain have in performing activities while in pain.^27^ The respondent considers how confident they are performing each activity, while taking their pain into account (0 not at all confident to 6 completely confident). A total score is calculated (ranging from 0 to 60) where higher scores indicate more confidence.

### Statistical analysis

We summarized continuous variables with means, standard deviations and T-scores. Dichotomous variables were summarized with percentages. Prior to do regression analyses, we assessed whether the data violated the formal requirements using appropriate methods. Using univariate analyses, we investigated the relationship between the CSS, the seven domains of the PROMIS-29, SF-MPQ-2, PCS, EQ-5D-5L, PSEQ (independent variables), and the extent of resilience measured with the CD-RISC (dependent variable). Based on this univariate assessment, we selected those domains showing statistically significant association with the outcome. In exploratory analyses using multivariate regression models, we investigated the relationship between the PCS and the sum of the significant PROMIS-29 domains and the CD-RISC. Using two thresholds from the literature for CD-RISC values, we classified groups of healthy subjects, primary care patients and generalized anxiety patients.^19^ For these three groups, we plotted the fitted corresponding PROMIS-29 sum values along with the 95% confidence interval. In another exploratory analysis, we assessed whether patients fully able to work had different CSS and resilience scores than those not able to work using a logistic regression model. *P* values <.05 were considered as statistically significant. Statistical analyses were performed using Stata, Version 16.1 (StataCorp LLC, College Station, Texas, USA). Study data were stored and managed with REDCap versions 6.12.1 to 6.14.1 (REDCap, Vanderbilt University, Nashville, TN, USA).

## Results

### Characteristics of the study population

We screened 79 patients with CRPS for our cross-sectional study, wherein five patients decided not to participate without giving further reasons. Therefore, 74 participants were recruited for this study. Three patients did not fill out the questionnaires, and ultimately 71 patients were enrolled in this study.

The majority of the participants were females (90.1%), and the mean age was 51.2 ± 12.9 years. The hand was affected almost twice as often as the foot and fractures represented the most common initiating event. The median duration of symptoms was 10 ± 26.6 months. More than half of the participants were not able to work due to their CRPS. The mean of the CD-RISC score was 70.9 ± 14.0. Compared to the reference scores reported in the validation study of the CD-RISC, this score was lower than in the US general population (80.4 ± 12.8) and among primary care patients (71.8 ± 18.4) and higher than patients with generalized anxiety disorders (62.4 ± 10.7).^19^ It should be noted that a low score on the CD-RISC suggests lower levels of resilience. The results of the PROMIS-29 questionnaire indicated mild (domains depression, fatigue, sleep disturbance, pain interference) to moderate symptoms (anxiety), a moderate impairment of physical function and no restriction in social participation.^28^ The characteristics of the study population are summarized in Table 1 and the results of the self-reported outcome variables are depicted in Table 2.

**Table 1.** Patient demographics (n = 71)

Characteristic	Variable
Gender (%)	
Male	9 (9.9)
Female	62 (90.1)
Age, years (mean, SD)	51.2 (12.9)
Location (%)	
Hand	44 (62)
Foot	27 (38)
Initiating event	
Fracture (%)	35 (49.3)
Bruise (%)	3 (4.2)
Strain (%)	9 (12.7)
Laceration (%)	3 (4.2)
Surgery (%)	21 (29.6)
Symptom duration in months (median, IQR)	10.0 (26.5)
Work status^a^	
Fully able to work (%)	17 (23.9)
Partially able to work (%)	14 (19.8)
Not able to work (%)	40 (56.3)

a related to CRPS.

SD = Standard deviation; IQR = Interquartile range.

**Table 2.** Self-reported outcome variables

Characteristic	Variable
CD-RISC mean (SD)	70.9 (14.0)
CSS, mean (SD)	11.7 (3.6)
SF-MPQ-2, mean (SD)	26.9 (13.2)
PCS, mean (SD)	19.7 (12.5)
EQ-5D-5L, mean (SD)	0.59 (0.23)
EQ-5D-5L VAS, mean (SD)	54.2 (22.5)
PSEQ, mean (SD)	35.6 (15.6)
Promis-29^a^	
Physical function (T-Score, CI)	38.2 (37.0–39.6)
Anxiety (T-Score, CI)	60.2 (58.0–62.5)
Depression (T-Score, CI)	59.4 (57.4–61.3)
Fatigue (T-Score, CI)	57.5 (54.7–60.3)
Sleep disturbance (T-Score, CI)	56.5 (55.6–57.5)
Pain interference (T-Score, CI)	56.2 (54.3–58.1)
Social participation (T-Score, CI)	49.7 (47.8–51.7)
Pain on VAS (last 7 days), mean (SD)	6.1 (2.2)

a Including suicide question.

SD = Standard deviation; CD-RISC = Connor-Davidson Resilience Scale (sum score 0–100, and higher scores indicate higher resilience); Promis-29 = Patient Reported Outcomes Measurement Information System 29-item Health Profile; CS = CRPS Severity Score; VAS = Visual Analogue Scale; PCS = Pain Catastrophizing Scale; EQ-5D-5L = EuroQoL 5-dimension 5-level instrument; SF-MPQ-2 = Short-form McGill Pain Questionnaire; PSEQ = Pain Self-Efficacy Questionnaire.

### Univariate analyses

**Figure 1.**
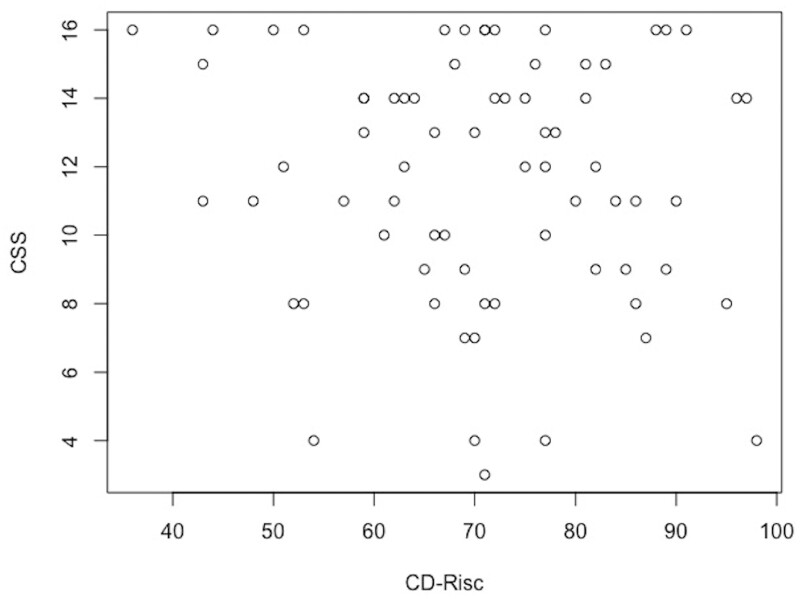
Association between the level of resilience and disease activity. CD-Risc = Connor-Davidson Resilience Scale; CSS = CRPS Severity Score

There was no association between CRPS severity and the level of resilience (-0.03 (95%CI: -0.09 to 0.03); *P* = 0.344) (Figure 1). Quality of Life as measured with the EQ-5D-5L was positively correlated with resilience (0.005 (95% CI: 0.001 to 0.009); *P* = .006), as was pain self-efficacy as measured with the PSEQ (0.35 (95% CI: 0.10 to 0.61); *P* = 007). The level of catastrophizing as measured with the PCS was inversely correlated with the level of resilience (−0.45 (95% CI: −0.63 to −0.26; *P* < .001). For the PROMIS-29 the domains of anxiety (−0.29 (95% CI: −0.44 to −0.15): *P* < .001), depression (−0.27 (95% CI: −0.40 to −0.15); *P* < .001) and fatigue (−0.39 (95% CI: −0.57 to −0.21); *P* < .001) were inversely correlated with the level of resilience. The domains of physical function, sleep disturbance, pain interference and social participation were not significantly correlated with the level of resilience.

### Exploratory analysis

**Figure 2.**
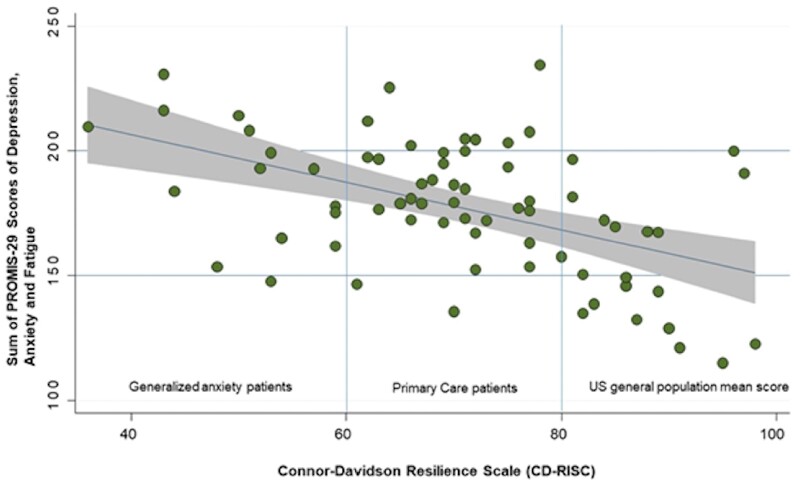
Association between the sum of Promis-29 scores and the level of resilience. The plot shows the regression line and the corresponding 95% confidence band. The blue lines specify cut-off values of CD-RISC for different groups of subjects according to the validation study of Connor et al.^19^

In an exploratory analysis we added the three statistically significant domains of the PROMIS-29 questionnaire (anxiety, depression, and fatigue) to a summary score and arbitrarily categorized it into three groups (<150 [low], <200 [intermediate], <250 [high]). Second, based on the reference scores from the validation study, we categorized the degree of resilience into three categories: (CD-RISC ≥80) corresponding to the US general population, intermediate resilience, (CD-RISC >60, <80) corresponding to primary care patients, and low degree resilience (CD-RISC ≤ 60) found in patients with generalized anxiety.^19^ In a logistic regression analysis, the proportion of patients with low degree of resilience increased with higher levels of anxiety, depression, and fatigue (odds ratio 2.65 (95% CI: 0.96 to 7.33); *P* = .061) with a low goodness-of-fit (Pseudo R^2^=5.2%) and without reaching statistical significance (Figure 2). Finally, we did not find a consistent pattern of CSS and CD-RISC scores between patients fully able to work and those unable to work.

## Discussion

### Main findings

The aim of this cross-sectional cohort study was to assess the degree of resilience in patients with CRPS 1, to explore the relationship between resilience and patient-related outcome measurements and to describe a pattern of clinical manifestations associated with low resilience. We found an association between quality-of-life pain, efficacy, and in selected domains of general health including anxiety, depression and fatigue, and the level of resilience. Interestingly, disease severity was not associated with the degree of resilience, indicating that the two concepts are not related. This indicates that, independent from disease activity, patients with CRPS with a high degree of anxiety, depression and fatigue should be screened for low resilience. Furthermore, we found no association between the level of resilience and severity of CRPS. This finding suggests that the severity of CRPS may be predominantly influenced by biological factors. This is an important consideration when developing treatment approaches for CRPS, as it underscores the need for interventions that address the underlying biological mechanisms of the condition.

### Results in the light of the existing literature

Resilience can be conceptualized when one is in pain as being able to recover from disability and depression, and sustaining functioning involving the ability to adapt to adversity.^29^ Resilience is not the only factor that influences coping response. Other factors include pain episode itself and vulnerability mechanisms (eg, catastrophizing, negative affect, or negative social interactions).^30^ Resilience is a dynamic process encompassing positive adaptation in the face of adverse experiences that would otherwise lead to poor outcomes.^31–33^ Resilience may not always result in recovery from disability but may also influence adjustment to disability that foster sustained participation not only from their health impairment but also related to societal restrictions. A recent study proposed that resilience may be an important extension of the fear avoidance model.^34^ In the fear avoidance model, individual responses to pain—in particular pain catastrophizing—may lead to pain-related fear and avoidance.^35^^,^^36^ Over time, avoidance of potential painful movement result in disuse, disability, and depression, and ultimately a vicious cycle of ongoing pain. Resilience may mitigate catastrophizing and thus be associated with more active coping style.

To date, resilience as a potential resource in CRPS 1 patients has received little attention. In the only article on this topic so far, Bodde et al.^37^ assessed the relationship between resilience and outcome after amputation in 26 patients with CRPS 1 and compared the results with reference groups from the literature respectively a control group from their outpatient rehabilitation clinic. The mean CD-RISC score of 73.3 ± 11.7 was slightly higher than in our study (mean 70.9 ± 14.0). The results showed that patients after amputation because of CRPS 1 who have a higher resilience also have a higher quality of life and experience lower psychological distress. The authors concluded that resilience should be further explored in Rehabilitation Medicine research in general because it potentially represents an additional treatment option in rehabilitation care.

A current review summarizing the role of resilience in orthopedic patients, concluded that resilience may contribute to favorable mental health and physical function. The authors recommended clinicians incorporating the resilience assessment into clinical practice, to identify patients at risk for an unfavorable postoperative outcome.^38^

### Strengths and limitations

To the best of our knowledge, this is the first study to assess resilience in a general CRPS 1 population. The strength of this study includes a well characterized cohort of CRPS 1 patients according to current international recommendations.^17^

The limitations of this study are fourfold. First, the relatively small sample size and the recruitment from a single center may have affected the results. However, the demographic and clinical characteristics of our study population are in line with the results of lager epidemiologic studies. Second, the cross-sectional design impeded our ability to capture the dynamics of resilience in the course of the disease and to explore potential causal relationships between the degree of resilience and the independent outcome parameters. Hence, a longitudinal study is needed to explore causality and the temporal sequencing of these findings. Third, it is unclear whether resilience can be influenced and thus, outcomes improved. Finally, the study was conducted before and during the global COVID-19 pandemic. This potentially have influenced resilience in some patients. The COVID-19 pandemic in Switzerland occurred in early February 2021 and the single lockdown lasted from March 16, 2021, to April 26, 2021. Since we included most participants before February 2021 (n = 53, 75%) and less than 10% (n = 7) were added after the lockdown, we do not expect the results were substantially influenced by the COVID-19 pandemic.

### Implication for practice

Coping strategies in patients with CRPS 1 may be important factors to consider. As disease severity seems to be unassociated with the degree of resilience, the assessment for low resilience in CRPS 1 patients should be targeted at those patients with a high degree of anxiety, depression, and fatigue rather than those with better quality of life or pain self-efficacy. We are unaware of a study showing the benefit of resilience training and positive clinical outcomes in CRPS 1. A meta-analysis on the efficacy of interventions to improve resilience found only weak evidence.^11^ Some single studies showed that specific interventions to improve resilience in patients undergoing hip fracture surgery and patients with diabetes have positive effects.^39^^,^^40^

### Implication for research

In our view two streams of research are needed. First, we need to increase our understanding regarding the dynamics of resilience in the clinical course of CRPS 1. This requires prospectively collected data for an extended period and several timepoints of re-assessment of health status, disease severity and resilience. Second, we need to understand if interventions targeted at increasing resilience have a positive effect on the course of CRPS 1 and to what extent specific subgroups gain greater benefits over others. Ideally, this would be assessed in randomized trials comparing a group receiving specific resilience training with a group receiving standard care alone.

### Conclusion

Resilience seems to be an independent factor in CRPS 1 and is associated with relevant parameters of the condition. Therefore, caretakers may screen the current resilience status of CRPS 1 patients to offer a supplementary treatment approach. Whether specific resilience training modifies CRPS 1 course, requires further investigations.
